# Central obesity and fat-free mass are associated with a larger spleen volume in the general population

**DOI:** 10.48101/ujms.v129.10465

**Published:** 2024-05-29

**Authors:** Mohammed Farah Mahmoud Mousa, Muhammad Naeem, Saima Bibi, Robin Bülow, Martin Bahls, Ulrike Siewert-Markus, Philipp Töpfer, Ali Aghdassi, Muhammad Nasir Khan Khattak, Henry Völzke, Marcello RP Markus, Till Ittermann

**Affiliations:** a Department of Study of Health in Pomerania/Clinical-Epidemiological Research, Institute for Community Medicine, University Medicine Greifswald, Greifswald, Germany; b Department for Radiology and Neuroradiology, University Medicine Greifswald, Greifswald, Germany; c Department of Internal Medicine B, University Medicine Greifswald, Greifswald, Germany; d German Centre for Cardiovascular Research (DZHK), partner site Greifswald, Greifswald, Germany; e Clinic and Polyclinic for Psychiatry and Psychotherapy, University Medicine Greifswald, Greifswald, Germany; f Department of Medical Psychology, University Medicine Greifswald, Germany; g Department of Medicine A, University Medicine Greifswald, Greifswald, Germany; h Department of Zoology, University of Malakand, Chakdara Dir (L), Pakistan

**Keywords:** Body characteristics, central obesity, Fat-free mass, MRI, spleen volume

## Abstract

**Background and aim:**

As the spleen plays a significant role in immunity, the aim was to investigate the associations of different body composition markers derived from various sources with spleen volume in a general population sample.

**Materials and methods:**

Cross-sectional data of 1095 individuals (570 women; 52%) aged between 30 and 90 years were collected in the Study of Health in Pomerania (SHIP-START-2). We measured spleen volume by magnetic resonance imaging (MRI).

Body composition markers were derived from classic anthropometry, bioelectrical impedance analysis, including absolute fat mass (FM) and fat-free mass (FFM), as well as from MRI, including visceral adipose tissue (VAT), subcutaneous adipose tissue (SAT), and liver fat content. Sex-stratified-adjusted linear regression models were used to analyze the associations of body composition markers with spleen volumes.

**Results:**

We observed positive associations of body mass index, body weight, waist circumference, hip circumference, waist-to-height ratio, absolute FM, absolute FFM, and VAT and SAT with spleen volume in men and women. An 8.12 kg higher absolute FFM was associated with a 38.4 mL (95% confidence interval [CI]: 26.7–50.1) higher spleen volume in men and a 5.21 kg higher absolute FFM with a 42.6 mL (95% CI: 26.2–59.0) higher spleen volume in women.

**Conclusion:**

Our findings indicate that obesity-related body composition markers and FFM are associated with a higher spleen volume. Particularly, higher absolute FFM showed a strong association with a larger spleen volume in both men and women. Further studies are warranted to understand the clinical significance of body composition markers on large spleen volume.

## Introduction

Currently, more than 650 million adults worldwide are patients with obesity, leading to increased morbidity, mortality, and healthcare costs (1). Total fat mass (FM) has received the greatest attention among the body’s constituents (water, lipids, protein, and minerals) (2). The assessment of metabolic status requires an evaluation of body composition and FM distribution (3). The etiology of chronic illnesses, such as type 2 diabetes, insulin resistance, cardiovascular diseases, and specific types of cancer, is strongly influenced by FM (4, 5). Waist circumference and abdominal obesity have been linked to inflammatory indicators in a stronger way than body mass index (BMI) or total FM (6).

Obesity causes low-grade inflammation (7) and is also considered a primary risk factor for metabolic syndrome (8). The spleen, as a largest lymphoid organ in the body, plays a significant role in immunity (9). Therefore, an interplay between obesity and splenectomy mediated by the immune system, as both are more prone to infections, is suggested (10). Another study supporting the above statement shows that individuals with metabolic syndrome have larger spleen size compared to those without metabolic syndrome (11).

Moreover, experimental studies on mice models also indicate a possible association between obesity and spleen size (12–16). For instance, in induced obese mice, a substantial increase (38%) was observed in spleen size (12). This suggests that central obesity might be a stronger risk factor for a larger spleen size compared to other anthropometric measures (13). These studies on induced mice models further reported that obesity suppresses the production of anti-inflammatory marker interleukin-10 in the spleen compared to normal mice, leading to inflammation in the liver and adipose tissues (15, 16). A similar increase in inflammation is also observed in splenectomy (14). Thus, animal studies suggest that obesity markers are associated with spleen volume, but the knowledge in humans is limited.

Only a few human studies investigated associations between body composition markers and spleen volume. For instance, a previous study found associations between waist circumference and spleen volume in a small and selected population (11). Similarly, three studies demonstrated associations of body height, body weight, and BMI with spleen volume in healthy volunteers and adolescents (17–19). However, in these studies, only indirect methods for measuring body composition were used (17–19). Similarly, little is known about different obesity marker associations with spleen volume in a general population.

Therefore, we aim to investigate the association of different body characteristics, derived from classic anthropometry, bioelectrical impedance analysis (BIA), and magnetic resonance imaging (MRI) with spleen volume using data from a large general population collected at the SHIP-START-2 cohort. The second aim of our study was to determine which of these markers show the strongest association with spleen volume.

## Material and methods

Analyses are based on data from the Study of Health in Pomerania (SHIP), which was conducted in Northeast Germany (20). In the present analyses, we used data from the second follow-up of SHIP-START (SHIP-START-2), in which 2333 individuals aged 30–93 years were examined between 2008 and 2012. At baseline, a sample from the population aged 20–79 years was drawn from population registries comprising 6267 eligible individuals. From them, 4,308 persons participated (response 68.8%) in SHIP-START-0. From the 2333 participants of SHIP-START-2, ten years after baseline, 1114 subjects were eligible and willing to undergo whole-body MRI. We excluded participants who underwent splenectomy (n = 3) as well as subjects with missing values for spleen volume (n = 16). The final analytical sample comprised 1095 participants (570 women; 52%) aged 30–90 years.

The study was conducted according to the guidelines laid down in the Declaration of Helsinki, and the protocol was approved by the Ethical Review Board of the University of Greifswald. All subjects gave their informed consent to be included in the study.

## Interview and physical examination

In standardized computer-assisted personal interviews, information on sex, age, smoking, and physical inactivity was collected. Smokers were categorized into three groups (lifetime non-smokers, former smokers, and current smokers). Individuals were classified as physically inactive if they reported less than one hour/week of exercise during summer and winter. Participants were asked to bring a list of all medications taken 7 days before the examination. Medication data were obtained online using the IDOM software (online drug database-led medication assessment) and categorized according to the Anatomical Therapeutical Chemical (ATC) classification index.

During the anthropometric examination, the subjects wore light clothing. BMI was calculated as weight in kilograms divided by the square of height in meters. Standardized measurements of height, weight, waist circumference, and hip circumference were performed. Body height was measured to the nearest 0.1 cm and weight to the nearest 0.1 kg by calibrated scales. Measurements of waist circumference and hip circumference were performed using an inelastic measuring tape with the subject standing comfortably with weight distributed evenly on both feet. Waist circumference was measured midway between the lower rib margin and the iliac crest in the horizontal plane. Hip circumference was determined as the greatest circumference between the highest point of the iliac crest and the crotch. Absolute FM and FFM were measured by BIA using a multifrequency Nutriguard-M device (Data Input, Pöcking, Germany) and the NUTRI4 software (Data Input, Pöcking, Germany) in participants without pacemakers (21). The electrodes were placed on the hand, wrist, ankle, and foot. The BIA uses three frequencies 5, 50, and 100 kHz, following the manufacturer’s instructions. Systolic and diastolic blood pressures were measured three times after an initial 5-min rest period on the right arm of seated individuals using a digital blood pressure monitor. Measurements were separated by 3-min intervals. The mean of the second and third measurements was calculated and used for the present analyses. Arterial hypertension was defined as elevated systolic or diastolic blood pressure ≥ 140/90 mmHg or intake of antihypertensive medication (ATC C02, C03, C04, C05, C07, C08, and C09). Participants were classified as having type 2 diabetes mellitus if they reported a physician’s diagnosis of disease in the interview took any glucose-lowering medications (ATC A10), or had elevated blood glucose or HbA1c levels (22). The estimated total blood volume (eTBV) was calculated by the Nadler’s formula (23).

## Magnetic Resonance Imaging

For the determination of visceral adipose tissue (VAT), subcutaneous adipose tissue (SAT), and liver fat content (LFC), MRI was performed using a 1.5-Tesla system (Magnetom Avanto, Siemens Healthcare AG, Erlangen, Germany, software version syngo MR B15). Abdominal fat was determined by axial 3D datasets using the 2-point Dixon technique (matrix: 256 × 176; slice thickness 4 mm/4 mm/3 mm without gap; 3 × 64 slices; inphase: TE 4.76 ms, TR 7.48 ms; opp-phase: TE 2.38 ms, TR 7.48 ms). The quantification of abdominal visceral and SAT was done using ATLAS (automatic tissue and labeling analysis software), an in-house developed software at the University of Ulm (24).

## Spleen volume assessment

Axial acquired diffusion-weighted MRI of the upper abdomen was performed on a 1.5-T MRI system (Magnetom Avanto; Siemens Medical Systems, Erlangen, Germany) using a 12-channel phased-array surface coil with subjects in a supine position. The isotropic diffusion-weighted imaging was performed using a spin-echo-based echo-planar imaging sequence. Imaging series with different diffusion weightings (b-values) were acquired using b-values of 50 mm^2^/s, 400 mm^2^/s, and 800 mm^2^/s. The acquisition was gated using a prospective acquisition correction technique and the following imaging parameters: repetition/echo time = 4140 / 72 [ms], field of view = 284 × 379 [mm^2^], a matrix of 192 × 115, a voxel size of 2.0 × 2.0 × 6.0 [mm], a slice gap of 1.2 [mm], a flip angle of 90°, and a bandwidth of 1735 Hz/Pixel. Quantitative image analysis of all b-800 images was performed by one observer after training, and inter-observer certification was computed together with a radiology resident with at least 5 years of abdominal MR imaging experience. Mean inter-observer variability was 1.30% (mean ± 1.96 standard deviations: -7.75 to 10.35; interclass correlation coefficient: 0.99) in a random subsample of 20 images. The volume calculation was performed by summation of each contoured spleen slice area with the slice thickness plus slice gap by MeVisLab®-Software (MeVis Medical Solutions AG, Bremen Germany) after the conversion of the acquired DICOM data to the Neuroimaging Informatics Technology Initiative standard (25, 26).

## Statistical methods

Continuous data are reported as mean and standard deviation (SD), and categorical data as absolute numbers and percentages stratified by quartiles of waist circumference. Multivariable linear regression analyses were used to associate body composition markers with spleen volume by calculating β coefficients and 95% confidence intervals (95% CI). To investigate the sex-specific effect on the association between various body characteristics and spleen volume, interaction terms of the respective exposure with sex were tested in the multivariable linear regression models.

All sex-stratified models were adjusted for age, physical inactivity, and smoking status. Models for the exposure weight, waist circumference, hip circumference, waist-to-hip ratio (WHR), SAT, VAT, and LFC were further adjusted for body height, while models for absolute FFM were adjusted additionally for absolute FM. To make the regression models comparable, all anthropometric markers were included as standardized variables. A *P*-value of *P* < 0.05 was considered to be statistically significant. All statistical analyses were performed using Stata 15.1 (Stata Corporation, College Station, TX, USA).

## Results

General characteristics of the study population are shown in Tables 1 and 2 for both men and women separately. The study population comprised 1095 individuals, of which 525 were men (48%) and 570 women (52%). Descriptive data of the study population were stratified by quartiles of BMI. Except for body height, the mean values of age, anthropometric, BIA, MRI markers, and eTBV increased from the first to the fourth quartile of BMI for both men and women. Higher spleen volumes were observed in the fourth quartile of BMI for both men and women. Considering smoking status, the majority of men in the fourth quartile were former smokers, while among women, the majority were never smokers in the third quartile of BMI. Moreover, both men and women in the fourth quartile had lower physical activity, more frequent type 2 diabetes, and were more likely to have type 2 diabetes and arterial hypertension compared to subjects in the other quartiles of BMI (Tables 1 and 2).

**Table 1:** Characteristics of the study population stratified by quartiles of body mass index in men (n = 543).

Parameter	First quartile N = (136)	Second quartile N = (137)	Third quartile N = (135)	Fourth quartile N = (135)
Age in years	53.4 ± 14.5	56.9 ± 13.9	57.2 ± 12.6	57.7 ± 11.5
Smoking in % Never Former Current	32.3% 46.3% 21.3%	27.7% 51.8% 20.4%	25.2% 53.3% 21.5%	23.9% 60.4% 15.7%
Body mass index in kg/m^2^	23.9 ± 1.33	26.5 ± 0.62	29.0 ± 0.83	33.0 ± 2.19
Body weight in kg	74.1 ± 6.67	82.0 ± 6.34	90.6 ± 7.41	101 ± 10.9
Body height in cm	176 ± 6.47	175 ± 6.07	176 ± 6.88	175 ± 6.76
Waist circumference in cm	85.9 ± 5.42	92.9 ± 5.23	99.4 ± 5.78	109 ± 7.98
Hip circumference in cm	94.6 ± 4.16	98.8 ± 4.30	103 ± 4.72	109 ± 6.65
Waist-to-hip ratio	0.91 ± 0.05	0.94 ± 0.05	0.97 ± 0.05	1.00 ± 0.05
Waist-to-height ratio	0.49 ± 0.03	0.52 ± 0.03	0.56 ± 0.03	0.62 ± 0.04
BIA				
Absolute FM in kg	15.0 ± 3.90	18.1 ± 3.58	22.5 ± 4.05	28.4 ± 6.48
Relative FM in %	20.2 ± 4.61	22.0 ± 3.85	24.8 ± 3.06	27.7 ± 4.48
Absolute FFM in kg	59.0 ± 5.60	63.9 ± 5.35	68.1 ± 6.09	73.6 ± 7.24
**MRI**				
VAT in L	3.63 ± 1.61	4.75 ± 1.76	6.50 ± 1.96	8.18 ± 2.41
SAT in L	4.44 ± 1.28	5.67 ± 1.32	7.42 ± 1.63	9.78 ± 2.43
LFC in %	4.40 ± 2.99	6.58 ± 4.66	8.16 ± 6.30	13.3 ± 8.74
Spleen volume in mL	190 ± 63.7	217 ± 97.2	235 ± 78.5	247 ± 92.7
eTBV in mL	4998 ± 413	5243 ± 407	5548 ± 466	5888 ± 544
Sedentary lifestyle in %	29.4%	27.0%	31.8%	33.6%
Type 2 diabetes in %	3.21%	7.83%	10.6%	19.2%
Hypertension in %	27.7%	44.0%	58.2%	73.5%

Data are expressed as mean and standard deviation for continuous data and as percentages for categorical data.

**Table 2:** Characteristics of the study population stratified by quartiles of body mass index in women (n = 584).

Parameter	First quartile N = (146)	Second quartile N = (146)	Third quartile N = (146)	Fourth quartile N = (146)
Age in years	51.2 ± 12.7	54.9 ± 12.2	57.0 ± 11.95	57.8 ± 11.4
Smoking in % Never Former Current	44.4% 34.7% 20.8%	45.0% 32.9% 21.3%	56.9% 27.4% 15.8%	48.6% 34.2% 17.1%
Body mass index in kg/m^2^	21.6 ± 1.35	25.0 ± 0.69	28.7 ± 0.99	34.1 ± 3.53
Body weight in kg	58.7 ± 5.59	66.8 ± 5.82	75.1 ± 6.77	89.1 ± 10.8
Body height in cm	164 ± 6.34	163 ± 6.59	163 ± 6.65	161 ± 7.1
Waist circumference in cm	71.9 ± 4.43	79.3 ± 5.48	87.4 ± 6.15	98.8 ± 8.93
Hip circumference in cm	91.3 ± 4.92	97.1 ± 4.71	104 ± 5.48	114 ± 6.65
Waist-to-hip ratio	0.78 ± 0.04	0.81 ± 0.05	0.84 ± 0.05	0.86 ± 0.05
Waist-to-height ratio	0.44 ± 0.03	0.48 ± 0.03	0.54 ± 0.04	0.61 ± 0.06
BIA				
Absolute FM in kg	15.7 ± 3.57	20.9 ± 3.72	26.9 ± 3.93	36.6 ± 7.20
Relative FM in %	26.6 ± 4.27	31.1 ± 3.59	35.8 ± 3.09	40.9 ± 3.79
Absolute FFM in kg	43.0 ± 5.24	45.9 ± 3.44	48.1 ± 4.12	52.3 ± 7.23
**MRI**				
VAT in L	1.31 ± 0.70	2.26 ± 1.04	3.31 ± 1.37	4.68 ± 1.57
SAT in L	5.17 ± 1.39	7.27 ± 1.39	9.54 ± 1.60	13.3 ± 2.84
LFC in %	2.72 ± 1.09	4.41 ± 3.96	5.88 ± 6.14	10.8 ± 8.93
Spleen volume in mL	147 ± 54.4	157 ± 54.2	168 ± 61.7	195 ± 71.7
eTBV in mL	3721 ± 346	3961 ± 538	4218 ± 403	4638 ± 504
Sedentary lifestyle in %	27.1%	21.4%	27.4%	34.9%
Type 2 diabetes in %	1.39 %	7.59%	8.22%	19.2%
Hypertension in %	26.1%	28.1%	49.3%	65.5%

Data are expressed as mean and standard deviation for continuous data and as percentages for categorical data.

**Figure 1:**
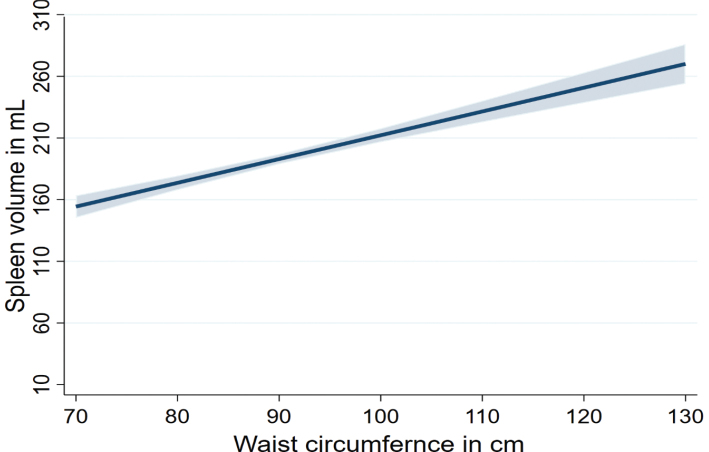
Associations of waist circumferences with spleen volume.

**Figure 2:**
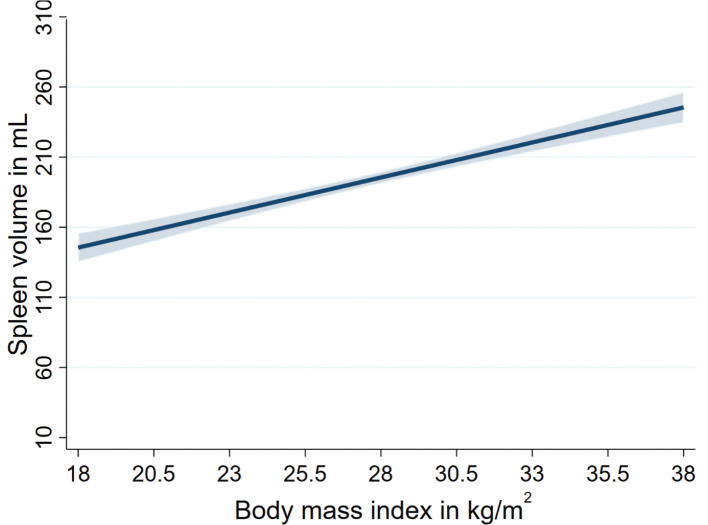
Associations of body mass indexes with spleen volume.

**Figure 3:**
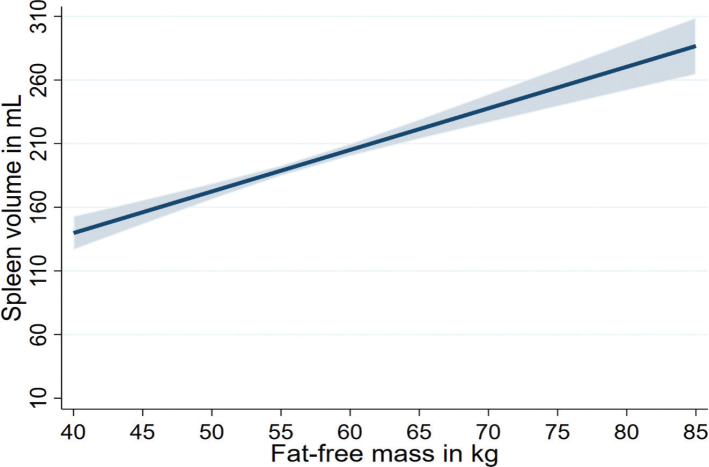
Associations of absolute FFM with spleen volume.

Multivariable linear regression stratified by sex and adjusted for age, smoking, and physical inactivity revealed significant associations between anthropometric markers and spleen volumes (Table 3). We found positive associations of standardized anthropometric, body fat distribution, and body composition markers with spleen volume in the whole population as well as in men and women (Figures 1 and 2). Of note, a 13.1 kg higher body weight was associated with a 33.3 mL (95% confidence interval [CI]: 24.0–42.5) higher spleen volume in men, and a 13.5 kg higher body weight was associated with a 24.8 mL (95% CI: 19.1–30.5) higher spleen volume in women. Similarly, a 8.12 kg higher absolute FFM was also associated with a 38.4 mL (95% CI: 26.7–50.1) higher spleen volume in men, and a 5.21 kg higher absolute FFM was associated with a 42.6 mL (95% CI: 26.2–59.0) higher spleen volume in women (Figure 3 and Table 3). In men, associations of BMI, body weight, waist circumference, hip circumference, WHR, absolute FM, and SAT with spleen volume were higher compared to women. However, the association of absolute FFM and VAT with spleen volume was higher in women compared to men. Overall, the associations of body composition markers with spleen volume were higher in men compared to women (Table 3).

**Table 3:** Adjusted* ß-coefficient (95%-CI) of the associations of standardized body compositions marker with spleen volume.

Exposure variables	Standard deviation	Men		Standard deviation	Women	
		β (95% CI)	*P*		β (95% CI)	*P*
Body mass index in SD	3.61 kg/m^2^	31.3 (22.9; 39.8)	<0.001	5.04 kg/m^2^	17.8 (13.3; 22.2)	<0.001
Body weight in SD	13.1 kg	33.3 (24.0; 42.5)	<0.001	13.5 kg	24.8 (19.1; 30.5)	<0.001
Body height in SD	6.54 cm	21.7 (10.7; 32.8)	<0.001	6.76 cm	16.5 (8.92; 24.1)	<0.001
Waist circumference in SD	10.6 cm	28.7 (19.8; 37.7)	<0.001	11.9 cm	21.9 (16.2; 27.6)	<0.001
Hip circumference in SD	7.31 cm	28.0 (18.5; 37.5)	<0.001	10.6 cm	16.5 (12.0; 20.9)	<0.001
Waist-to-hip ratio	0.06	22.0 (11.7; 32.3)	<0.001	0.06	16.7 (8.58; 24.8)	<0.001
Waist-to-height ratio	0.06	27.2 (18.3; 36.2)	<0.001	0.08	16.7 (11.5; 21.8)	<0.001
BIA						
Absolute FM in SD	6.84 kg	23.9 (15.2; 32.5)	<0.001	9.16 kg	19.2 (14.7; 23.7)	<0.001
Absolute FFM in SD	8.12 kg	38.4 (26.7; 50.1)	<0.001	5.21 kg	42.6 (26.2; 59.0)	<0.001
MRI						
VAT in SD	2.57 L	14.4 (6.63; 22.2)	<0.001	1.70 L	22.5 (13.6; 31.4)	<0.001
SAT in SD	2.59 L	20.7 (12.0; 29.3)	<0.001	3.46 L	13.4 (8.56; 18.3)	<0.001
LFC in SD	6.88 L	16.5 (8.60; 24.4)	<0.001	6.59 L	11.6 (5.57; 17.5)	<0.001

* Linear regression with standardized coefficients adjusted for age, smoking, and physical inactivity. Models for weight, waist circumference, hip circumference, waist-to-hip ratio, SAT, VAT, and LFC were further adjusted for body height, while for FFM, the model was further adjusted for absolute FM.

We investigated the effect of sex on the associations between different body composition markers and spleen volume (Table 4). We found only a positive sex-specific interaction of BMI and hip circumference with spleen volume. However, the other studied markers showed no statistically significant interaction with sex on spleen volume (Table 4).

**Table 4:** Adjusted* ß-coefficient (95%-CI) for the sex-specific interaction of standardized body compositions marker with spleen volume.

Variables	Men	Women	*P* for interaction
	β (95% CI)	β (95% CI)	
Body mass index in SD	30.4 (23.1; 37.7)	19.4 (14.2; 24.5)	**0.015**
Body weight in SD	31.3 (23.9; 38.7)	25.4 (18.7; 32.1)	**0.227**
Body height in SD	21.6 (12.3; 30.9)	16.4 (7.86; 25.0)	**0.398**
Waist circumference in SD	28.1 (20.5; 35.7)	22.6 (16.1; 29.1)	**0.261**
Hip circumference in SD	27.5 (19.6; 35.4)	16.3 (11.1; 21.5)	**0.017**
Waist-to-hip ratio in SD	20.4 (11.5; 29.3)	19.6 (10.4; 28.8)	**0.896**
Waist-to-height ratio in SD	26.3 (18.8; 33.9)	17.6 (11.8; 23.3)	**0.059**
**BIA**			
Absolute FM in SD	24.0 (16.5; 31.5)	19.0 (13.6; 24.4)	**0.285**
Absolute FFM in SD	38.0 (28.6; 47.3)	43.4 (26.6; 60.1)	**0.527**
**MRI**			
VAT in SD	17.2 (10.5; 23.9)	23.2 (13.0; 33.3)	**0.316**
SAT in SD	23.4 (15.8; 31.0)	13.8 (8.00; 19.6)	**0.050**
LFC in SD	17.1 (10.0; 24.2)	10.6 (3.51; 17.6)	**0.197**

* Linear regression with standardized coefficients adjusted for age, smoking, and physical inactivity. Models for weight, waist circumference, hip circumference, waist-to-hip ratio, SAT, VAT, and LFC were further adjusted for body height, while for absolute FFM, the model was further adjusted for absolute FM.

In sensitivity analysis, we found that absolute FFM was strongly correlated with the eTBV (r = 0.95), explaining 90% of the variation in eTBV (R^2^ = 0.90).

## Discussion

Limited studies are available on spleen size and its pathophysiological relevance in the general population. This is the first study investigating the associations of body composition with spleen volume in a general population. We observed associations of body fat markers and absolute FFM with spleen volume in 1095 German adults (men and women). Among all markers considered, the associations of body composition markers with spleen volume were slightly stronger in men compared to women.

All the body composition markers showed an association with the higher spleen volume in our study; however, the association tends to be higher considering ß-coefficient values. The associations of body composition markers with spleen volume were notably higher in men compared to women. Similar to our finding, a study including 400 healthy volunteers found a positive association of body height and weight with ultrasound-assessed spleen volume (19). Another large study also observed significant associations of body height and weight with spleen volume in both men and women (17). In line with this, a study conducted in patients (n = 160) showed only a significant difference between body height and spleen volume but not between body weight and spleen volume (27). In our study, we compared the effect sizes of various anthropometric markers with spleen volume in a large population-based sample. The associations of anthropometric markers, absolute FM, and SAT with spleen volume were slightly higher in men compared to women, while absolute FFM and VAT showed a slightly higher effect size with spleen volume in women compared to men. Furthermore, we used MRI for the determination of spleen volume, in contrast to the aforementioned previous studies that utilized ultrasound.

The underlying pathophysiological mechanism explaining the associations between body composition markers and spleen volume remains largely unknown. We believe that an increase in both eTBV and plasma volume, following an increase in absolute FFM, might provide an explanation (28). The skeletal muscle makes up approximately 48% of the body’s fat-free mass (29) and has a significant impact on changes in blood and plasma volumes due to its metabolic demands (30). A previous study indicated that FFM is responsible for about 90% of the variation in TBV and plasma volume (31). In sensitive analysis, we found that FFM explains 90% of the variation in eTBV. We believe that elevated eTBV and plasma levels lead to an increased volume load on the spleen, consequently leading to a larger spleen volume. Our previous study also found positive associations of spleen volume with hemoglobin and red blood cell count (26). Another mechanism that could explain our associations is inflammation. It is well-established that inflammation of adipose tissue is common in a patient with obesity (32). Additionally, the spleen plays an important role in inflammation, housing a large number of leucocytes (33). Therefore, we can hypothesize that obesity mediated by inflammation might be responsible for larger spleen volume in our study.

Our study has at least two limitations. First, the cross-sectional design does not allow us to conclude about causal inference and the direction of associations between body compositions and spleen volume. Second, in our study, we used BIA instead of dual-energy x-ray absorptiometry (DXA). While DXA is considered one of the most accurate methods for assessing FM and FFM, its ionizing nature makes it difficult to use in a general population-based sample (34).

The strength of our study is the large population-based study design and the use of various modalities like MRI for the assessment of body fat distribution and spleen volume. MRI is considered more sensitive and accurate for fat quantification in the body compared to ultrasound and CT (35). Additionally, we included several body composition markers derived from BIA.

## Conclusion

Our findings indicate that obesity-related body composition markers as well as FFM are associated with a larger spleen volume. Notably, higher levels of absolute FFM were strongly associated with larger spleen volume in both men and women. Further longitudinal studies are needed to understand the effect of body composition markers on larger spleen volume.

## Data Availability

The datasets generated and/or analyzed during the current study are available from the corresponding author upon reasonable request.
